# Full-Locked Coil Ropes with HDPE Sheath: Studies of Mechanical Behavior of HDPE Under Accelerated Aging

**DOI:** 10.3390/ma18010106

**Published:** 2024-12-30

**Authors:** Benjamin Schaaf, Björn Abeln, Markus Feldmann, Elisabeth Stammen, Klaus Dilger

**Affiliations:** 1 Institute of Steel Construction, RWTH Aachen University, 52074 Aachen, Germany; 2 Institute of Joining and Welding, Technical University of Braunschweig, 38106 Braunschweig, Germany

**Keywords:** HPDE, bridge cables, corrosion protection, accelerated aging, mechanical characterization

## Abstract

In accordance with German guideline ZTV-ING Part 4, full-locked coil ropes are provided with a three-layer corrosion protection coating based on epoxy resin and polyurethane, which must be renewed regularly. An alternative method is to use a coating of high-density polyethylene (HDPE), which is extruded onto the rope. In this article, the mechanical behavior of the thermoplastic material is studied, taking into account various accelerated aging processes, which are derived from the climatic boundary conditions of a real bridge structure and implemented in tests. In addition to the quasi-static material behavior, which is described using the uniaxial tensile test, the cyclic conditioning, relaxation, type of production and oxidation stability are also investigated. Finally, the results obtained are evaluated with regard to the applicability of the material as corrosion protection for full-locked coil ropes.

## 1. Introduction

**Figure 1:**
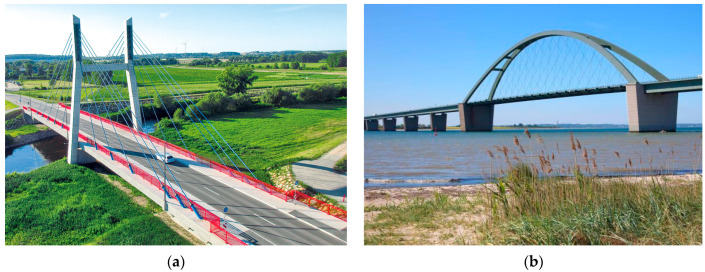
(**a**) Mulde Bridge Schlunzig (photo: Schulze + Rank Ingenieurgesellschaft); (**b**) Fehmarnsund Bridge (photo: dpa).

If spans of more than 150 m have to be overcome for traffic routes, cable-span bridges are an economical construction method in bridge building. Since the beginning of the 2000s, the construction of this type of bridge has increased significantly. A distinction can be made between suspension bridges, cable-stayed bridges, arch bridges and other bridge types. Cable-stayed bridges in particular are frequently used in Germany due to their design advantages, such as the absence of costly abutments [1]. For example, the new replacement Rhine bridges near Leverkusen and Duisburg-Neuenkamp are being built as cable-stayed bridges. Regardless of the type of bridge, cables play a central role as high-strength tension members for the load-bearing capacity of the bridge structure. The requirements for corrosion protection of the bridge cables are therefore correspondingly high. In accordance with the state of the art as per German guideline ZTV-ING Part 4 [2], three-layer corrosion protection systems based on epoxy resin and polyurethane are used, which are applied after the ropes have been installed. A new alternative is the use of HDPE sheathing, which is extruded onto the cable by the factory. This makes it possible to dispense with the need for subsequent applications of the corrosion protection coating in the structure and maintenance coatings during the service life. HDPE-coated fully locked cables were used for the first time on the Schlunzig Mulde bridge (Figure 1a).

The investigations presented here show that the HDPE coating is a competitive alternative to conventional corrosion protection coatings for full-locked coil ropes (FLCR) due to its resistance to climatic influences.

Experimental studies are discussed, considering various accelerated aging methods derived from the climatic requirements of the network arch bridge over the Fehmarnsund (Figure 1b).

### 1.1. Bridge Cables

#### 1.1.1. Conventional Bridge Cables

**Figure 2:**
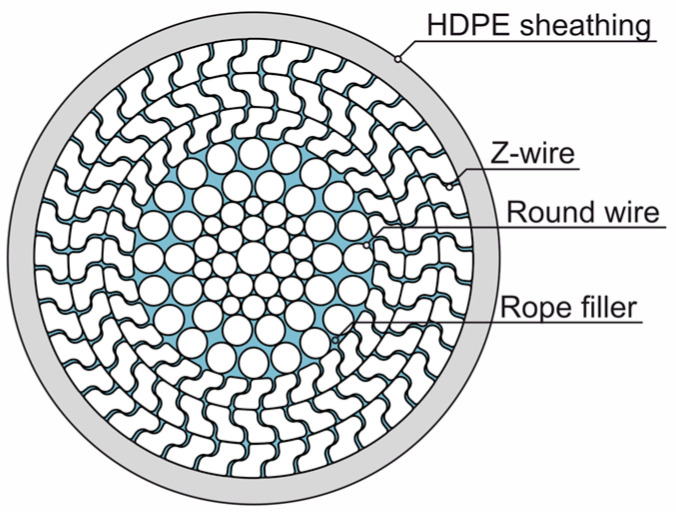
Technical illustration of full-locked coil rope (FLCR).

The most common type of bridge rope used in Germany are FLCRs. They consist of an inner core of round wires several layers thick and at least two outer layers of shaped wires (Z-wires), Figure 2. The first layer contains a core wire with six round wires laid in a helix. The other wire layers are stranded spirally in alternating directions [3]. Under tensile load, the interlocking Z-wires of the outer layers form a closed surface, which prevents the intrusion of corrosive media [1].

FLCRs have a total of three corrosion protection barriers. The first anti-corrosion barrier is the outer coating of the rope. The current construction method involves a multi-layer coating system with an epoxy resin-based primer. Several polyurethane-based functional layers are applied on top of this.

With the exception of the primer coat, the coating system must only be applied after installation in accordance with ZTV-ING [2], which requires the use of scaffolding or appropriate coating robots [4]. A rope filler (lube) is inserted into the rope to prevent internal friction and as additional corrosion protection (the second corrosion protection barrier). Only hot-dip galvanized wires may be used as an internal corrosion protection barrier.

In Germany, bridge cables are designed in accordance with DIN EN 1993 Part 1-11 [5] and the associated national annex. Technical test specifications and delivery conditions for fully locked coil ropes are regulated within the TL/TP-VVS [6].

#### 1.1.2. FLCR Sheathed with HDPE

**Figure 3:**
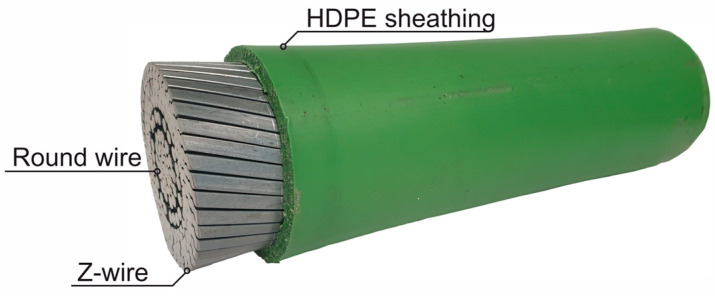
Sample of the new cables of the Fehmarnsund Bridge with green HDPE sheathing.

The use of an FLCR with an HDPE sheath offers a new and economical alternative to conventional FLCR with an anti-corrosion coating. A major advantage is the complete production of the outer corrosion protection barrier in the factory by means of extrusion, which eliminates the need to apply the corrosion protection after the bridge cables have been installed, as is the case with conventional FLCRs. The HDPE sheath is extruded onto the bridge cable at approx. 160 °C after stranding. The thickness of the HDPE sheath is usually 4 to 7 mm. A wide range of colors can also be selected using appropriate additives. Figure 3 shows a sample of the new bridge cables of the Fehmarnsund Bridge in the characteristic green color of the original cables.

The specifications for HDPE-coated bridge cables can be found in fib Bulletin 30 [7].

On the one hand, requirements are made for the material composition, such as density, carbon black content or thermal stability. On the other hand, minimum requirements are specified with regard to mechanical characteristics such as modulus of elasticity, strength and fracture strain. These are differentiated into requirements relating to the raw material and the extruded pipe. All mechanical tests are carried out using uniaxial tensile tests on the dog bone specimen in accordance with ISO 527-2 [8]. In Germany, the use of HDPE-coated FLCR requires approval from building authorities [1].

**Figure 4:**
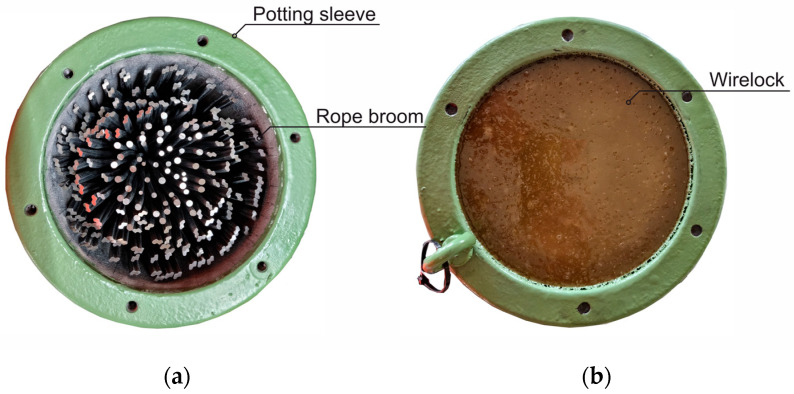
(**a**) Cable sleeve prepared for socketing with fanned-out cable broom; (**b**) cable head cast with WIRELOCK^®^.

To anchor the FLCR, the rope ends are formed into a uniform rope broom and placed in a tapered rope sleeve. If FLCRs with HDPE sheathing are used, the anchorage of the cable broom is not to be made with a metal filling as usual, but with a synthetic resin filling (Figure 4a).

According to TL/TP-VVS [6], the rope sleeves must be cast with the alloy “Zamak Z 610”, which consists mainly of zinc, whereby the casting takes place at approx. 450 °C and the cable head is heated to 350 °C. However, the melting point of HDPE ranges from approx. 120 °C to 160 °C, meaning that the Zamak casting would melt or thermally decompose the HDPE, which would be equivalent to destroying the corrosion protection. In addition, HDPE softens considerably in temperature ranges below the melting point, which would lead to inhomogeneities in the HDPE sheath in cooler areas away from the cable head.

For this reason, socketing with a synthetic resin filling is used, Figure 4. These are usually polyester or epoxy resins. WIRELOCK^®^, a two-component polyester resin with a quartz powder as filler with approx. 49% by mass, is an established product on the market [9]. The socketing compound hardens within a short time, developing heat up to approx. 120 °C. The heat can be dissipated quickly via the potting sleeve and the rope. Requirements for synthetic resin potting for steel wire ropes can be found in DIN EN 13411-4 [10].

The use of HDPE-coated FLCRs has hardly been widespread in Germany to date. This type of FLCR was used for the first time on the Schlunzig Mulde Bridge (Figure 1b). In the course of exchanging the cables of the Fehmarnsund Bridge, FLCRs with HDPE sheathing and synthetic resin grouting were used. This type of bridge cable is already more established internationally, for example on the Strömsund Bridge in Sweden and the BØkfjord Brigde in Norway, as well as some bridges in the Far East and the USA.

### 1.2. High-Density Polyethylene

#### 1.2.1. Basic Properties

High-density polyethylene (HDPE) is a thermoplastic polymer material produced by the polymerization of ethylene on certain catalysts in a medium to low pressure range. The high density of HDPE (more than 0.94 g/cm^3^) results from a low degree of visualization of its molecular structure and the resulting increased crystallinity, which leads to improved strength and rigidity. These properties make HDPE particularly suitable for applications where structural integrity and durability are required [11].

HDPE is also known for its resistance to a wide range of aggressive chemicals, acids and bases. UV stabilizers and antioxidants can be added during production to increase UV and oxidation stability [12]. The material is particularly attractive for use in chemically explosive environments.

The crystallinity of HDPE is typically in the range of 60–80%. With increasing crystallinity, the modulus of elasticity, tensile strength, hardness, density and melting temperature also increase, while elongation decreases. Extruded HDPE has a modulus of elasticity of approx. 800–1000 MPa in the linear-elastic range. The strength is usually in the range of 20–25 MPa. HDPE generally has a pronounced yield potential, with a correspondingly high fracture strain in the range of 300–600%. Depending on the manufacturing process, however, the yield potential can be considerably reduced. As for most plastics, a dependence of the mechanical parameters on the loading rate and temperature can be observed [13]. Their influence on the crystallinity, density, tensile strength and modulus of elasticity, among other things, is determined by the superstructure of the semi-crystalline HDPE. In addition to the amorphous tangle structures of the polymer chains, the crystallites form first orders lamellae and later spherulites. With slow cooling in the manufacturing process, fewer spherulites are formed, but these can become comparatively large, whereas with rapid cooling only very small spherulites are formed [14]. The manufacturing process can therefore influence the material properties and must therefore be taken into account when testing the material properties. As described by Chaoui et al. in [15], the extrusion process for HDPE pipes is responsible for a multitude of morphological and intrinsic heterogeneities through the pipe wall. The fast quenching of the outer wall and the slow cooling at the inner surface leads to a gradient of stresses, (semi-)crystallinity and content of antioxidants.

In the project, HDPE material was provided by the manufacturer, which was taken directly from the rope sheaths, and that represents a complete cross-section through the extruded mass. The following material characteristics were taken from the supplied quality assurance protocols:

- The impact resistances are 14.2 kJ/m^2^ from the unnotched Charpy test according to ISO 179-1 [16], with an elongation at break of 136% at −20 ± 2 °C and 50 mm/min (ISO 6259-1 and -3 [17,18]);
- The bulk density is 0.958 g/cm^3^ in accordance with ASTM D1505 [19]. The melt flow index according to ASTM D1238 [20] is 0.051 gm/10 min.

Due to the small number of samples provided, these values could not be tested and were adopted.

#### 1.2.2. Resistance of HDPE

HDPE has excellent resistance to a wide range of influences. For use as a corrosion protection and sealing barrier for the FLCRs of bridge structures, it is exposed to weathering. The relevant climatic influences are thus temperature changes, water and moisture, in a maritime environment or near roads, saline solutions and UV radiation. In addition, there are dynamic loads due to traffic loads and wind effects.

Albozahid et al. [21] studied the influence of moisture and UV radiation. Moisture leads to a slight degradation of the modulus of elasticity and strength. Sunlight or UV radiation from artificial sources lead to chain defects and thus also to a reduction in mechanical properties. The aging of HDPE bottles in sea water for up to 72 days at ambient and high temperature (80 °C) by Elkori et al. [22] showed similar results.

Hsueh et al. [23] and Carrasco et al. [24] observed an embrittlement of the material under UV irradiation (an increase in modulus, reduction in fracture strain). The higher the temperature prevailing during irradiation, the greater the embrittlement. Zhao et al. [25] found in thermal oxidation tests that the tensile strength increases with the aging time, while the elongation first increases (by specific cross-linking reactions) and then decreases.

There have been extensive studies on the aging behavior of HDPE, particularly with regard to pipelines or geomembranes. Mueller and Jakob [26] showed changes in the mechanical properties and oxidative induction times (OITs) of HDPE geomembranes during aging behavior in hot air and hot water, where oxidation is essentially dependent on the slow loss of antioxidants. The oxidation starts only after the depletion of antioxidants and then leads quickly to the brittleness of the sample, but no complete oxidative deterioration has been observed. Ewais and Rowe [27,28] have studied HDPE geomembranes in leachate at different temperatures, and morphological changes were observed before the antioxidants were depleted. Guermazi et al. [29] have investigated the accelerated aging of HDPE coatings in marine applications under the influence of seawater at different temperatures. A change in the mechanical properties was found, which depends on the aging temperature and the aging cycle.

In bridge construction, HDPE has only been used occasionally to protect bridge cables against corrosion.

Friedrich [4] compared the advantages and disadvantages of fully sealed spiral cables and strand bundle cables for cable-stayed bridges as a decision-making aid for future planning. Only the classic corrosion protection and butyl cheek bands were considered, not HDPE sheathing. Aging tests were not used for the comparison.

Saul and Nützel [30] investigated automatic wrapping with butyl bonding tapes as an alternative to the conventional coating of fully sealed bridge cables for the refurbishment of a suspension bridge in Norway. The focus was on the development of the process; no aging tests were carried out and no comparison to HDPE.

Yu et al. [31] evaluated the corrosion of steel cables using accelerated corrosion tests on semi-parallel cables with different HDPE sheath breaks. The focus here was on the corrosion of the steel, not the aging of the HDPE.

Huang et al. [32] examined the fatigue behavior of HDPE sheathing with regard to mechanical behavior (friction losses, slip, wear resistance).

Liu et al. [33] analyzed HDPE jackets using small part samples that were subjected to up to 5000 h of accelerated aging with a xenon arc lamp. The focus here was on the change in mechanical behavior. A critical time was determined, after which the mechanical characteristics decrease and the service life is reduced. No extrapolation was made to a specific service life, and the aging was carried out solely under xenon illumination.

Mkacher et al. [34] have presented a general methodology for assessing the durability of HDPE outer jackets. Their investigations are based on a series of experiments to clarify aging mechanisms by means of thermal oxidation tests on stabilized and pure PE, the determination of the chemical consumption of antioxidants and the formation of oxidation products.

Lin and Zhang [35] examined cable sheaths made of HDPE in a dry environment and carried out an artificial aging test in daylight and a tensile test under different load levels. The results showed that as the HDPE jacket aged, damage gradually occurred and the mechanical properties of the HDPE jacket also decreased.

Dynamic permanent loads influence the yield elongation and stress as well as the modulus of elasticity of HDPE [32,36,37]. However, the strain amplitudes required for this must be relatively large in order to achieve significant changes. In addition, HDPE exhibits time-dependent (viscoelastic) material behavior. Lai und Bakker [38] studied the creep behavior of HDPE and observed non-linear creep. With the help of a time–tension superposition, accelerated statements can be made about long-term creep. In addition, retardation spectra are shifted towards longer periods of time due to physical aging. A rate dependence of the material was experimentally proven by Elleuch and Taktak [12] for compressive and tensile loads.

The previous investigations show the influence of different aging effects on the material HDPE by (UV) light, oxygen and temperature and loads. For the application of FLCRs, the accelerated aging processes described in Section 2.2. are derived using the Fehmarnsund Bridge, which is very exposed to the climate in a maritime environment. Compatibility with the various substances with which the HDPE pipe comes into contact during its service life is also analyzed. The influences of hydrocarbon compounds like crude oil or motor oils and fuels on HDPE were tested by Chaoui et al. [15] and Rezakalla and Gennadyevech [39]. Within the first weeks, a decrease in modulus and strength became obvious. Due to this major influence, engine oil is taken into account within our analyses.

## 2. Materials and Methods

### 2.1. High-Density Polyethylene

**Figure 5:**
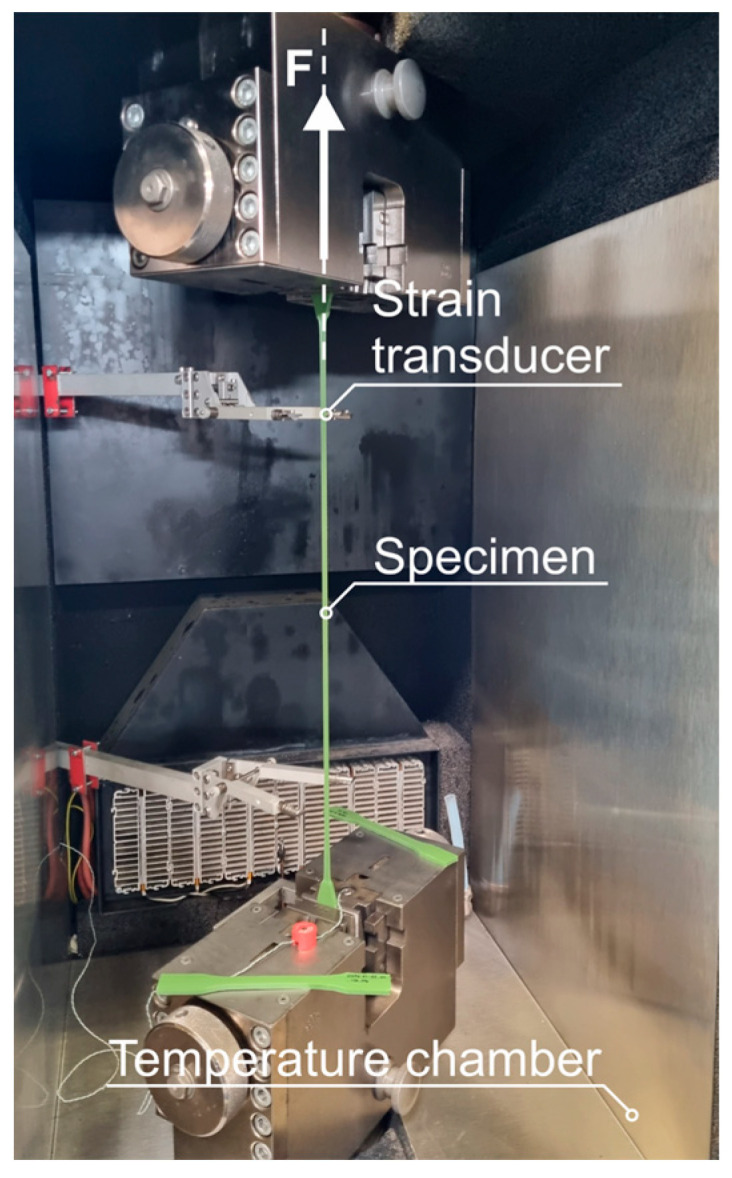
Tensile test according to DIN EN ISO 527 [8] using a temperature chamber.

The influence of artificial aging, as well as the compatibility with other materials and temperature dependence, are primarily studied on the basis of the change in mechanical behavior under tensile load. Based on fib Bulletin 30 [7], tensile tests are carried out in accordance with DIN EN ISO 527-2 [8] on test specimen type 1B (Figure 5).

The tests are carried out on a uniaxial *Zwick Roell Z100*, ZwickRoell GmbH and Co. KG, Ulm, Germany, testing machine. Deformation quantities are recorded locally in the parallel measuring area of the shoulder specimen using two longitudinal extensometers. These are also used for displacement-controlled regulation at 50 mm/min in accordance with [7]. A preload of 10 N is applied prior to testing.

The characteristic values of strength, fracture strain and modulus of elasticity are determined on the basis of the experimentally collected data. The latter is determined in the stress interval [1 MPa; 4 MPa]. This range is particularly suitable due to the relatively linear behavior of the material. The corresponding strains are between 0.5% and 0.25%, an interval that is usually used to determine the modulus.

### 2.2. Accelerated Aging Procedures

#### 2.2.1. Sanding Method

Due to the location of the Fehmarnsund Bridge, the HDPE coating of the bridge cables is constantly abraded by sand during its service life, which is carried to the bridge from the surrounding beaches and shore areas, especially in strong winds (sand abrasion).

**Figure 6:**
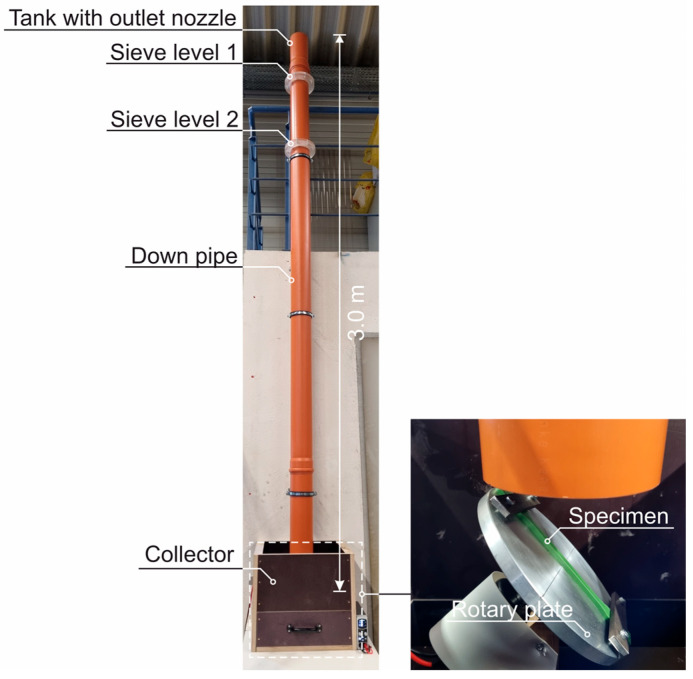
Sanding device based on DIN 52348 [40].

Selected HDPE samples are spattered with sand for testing. The sanding method for glass and plastics according to DIN 52348 [40] is used for this purpose. To increase the abrasion of the HDPE, the test is carried out at twice the drop height of 3.0 m (Figure 6). A total of 3 kg of quartz sand (grain size 0.5/0.71) falls evenly through a special outlet nozzle and two sieve levels onto the sample, which is mounted on a rotary plate to ensure homogeneous abrasion.

#### 2.2.2. Climatic Boundary Conditions and Compatibility

**Figure 7:**
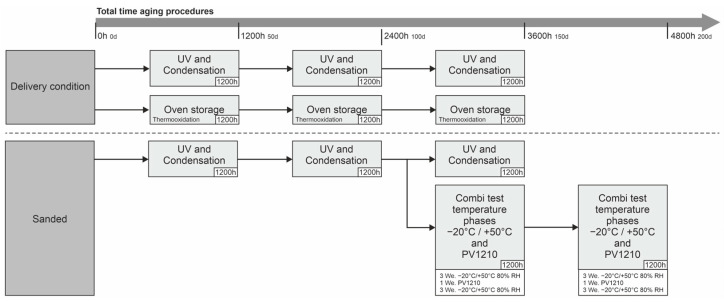
Overview of the accelerated aging procedures carried out.

Three basic types of climatic exposure are analyzed as part of the accelerated aging process: temperature cycles, salt spray and UV irradiation. The exposure is combined in order to consider possible, mutually reinforcing influences on the mechanical properties of the HDPE. The combinations of the aging processes carried out can be seen in Figure 7.

The selected temperature range of −20 °C to +50 °C corresponds to the extreme temperatures to be expected at the Fehmarnsund Bridge. Due to the green color of the bridge cables and the maritime climate of the Baltic Sea, a surface temperature of more than +50 °C is not to be expected even on sunny summer days. The temperature range of −20 °C to +50 °C is used as the upper and lower test temperature for both the accelerated aging methods and the uniaxial tensile test. The upper service temperature of HDPE without mechanical stress should not exceed 90 °C to 100 °C for short periods and 80 °C to 90 °C for long periods [11].

To investigate the influence of UV radiation on the material, aging tests are carried out with artificial irradiation in accordance with DIN EN ISO 4892 Part 1 [41] and Part 3 [42] using a *UVTest^®^* by Atlas Material Testing Technology GmbH, Linsengericht, Germany. Cycle 1 of procedure A with a UVA-340 lamp is selected. This contains drying and condensation phases.

As already discussed, a correlation between real and accelerated aging for plastics and HDPE in particular has not been described or cannot be determined experimentally for the most part due to a large number of simultaneously acting influences. With regard to UV exposure, however, a comparative calculation can be used to make a rough estimate of the artificial UV irradiation and the UV exposure occurring on the building. Using data from the German Weather Service (DWD), the average annual sum of global radiation on Fehmarn can be determined as 1040 kWh/m^2^. According to DIN EN ISO 4892-1 [41] the proportion of UV radiation is approx. 6.8% of the total radiation. With the power of the UV source (0.76 W/m^2^), a duration of approx. 39 days corresponds to the real UV exposure of a year on Fehmarn. If it is also taken into account that the UV lamp is switched off according to cycle 1, method A, during the wet phases, the duration is extended to approx. 50 days. The total duration of the artificial irradiation of 3 × 50 days thus corresponds approximately to a UV exposure of three years. It must also be taken into account that the constant change between wet and dry phases puts more strain on the material than in a constantly dry climate.

Samples are collected after every 50 days (1200 h) and tested in a uniaxial tensile test. In addition, further samples are collected at one-week intervals in order to be able to analyze possible degradation with a higher resolution.

**Figure 8:**
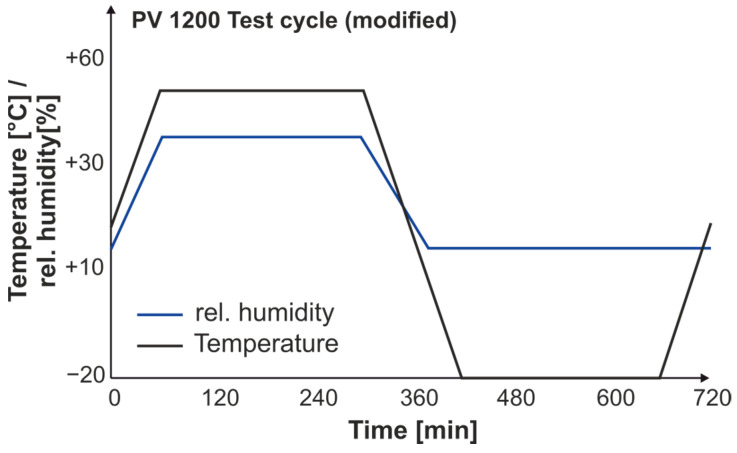
PV1200 test cycle.

After 2400 h of UV irradiation, some samples are taken and subjected to a corrosion and aging test. This consists of the modified aging test PV1200 (−20 °C/+50 °C with 80% RH) in a *C600-70* climate chamber by Weiss Technik GmbH, Reiskirchen, Germany and the condensation and salt spray test PV1210 (both tests according to VW AG specifications) in a *SC/KWT 1000* salt spray chamber by Weiss Technik GmbH, Reiskirchen,, Germany. A 1200 h block is constructed according to the following aging regime. A detailed description of the two aging tests can be found in Table 1 and Figure 8. The modified aging test is also extended with additional samples in order to resolve the degradation of the mechanical properties more precisely. Details can be found in the general overview in Table 2.

In addition to the climatic influences, the compatibility of the HDPE with rope filler and mineral oil is also analyzed. For this purpose, samples are stored fully enclosed in both media at room temperature for 1200 h (50 days). The filling agent is a solvent-based rope lubricant containing zinc dust. Table 2 summarizes the descriptions and properties of all the sample series studied.

#### 2.2.3. Cyclical Conditioning

Some plastics exhibit material softening under repeated loading. This can manifest itself in the form of a reduced stiffness after initial loading (Mullins effect) or in the form of reduced strength.

During the manufacturing and installation of the bridge cables, the HDPE sheath is also subjected to repeated cyclical stress. The rope is coiled up after the sheathing has been extruded, uncoiled before the rope core is socketed, coiled up again after socketing and finally uncoiled during installation. With a winding diameter of 2.20 m, this results in a maximum compression of approx. 3.1% on the inside and maximum elongation of approx. 3.0% on the outside of the HDPE sheath.

In order to investigate the influence of this load cycle, uniaxial tensile tests are carried out after a defined cyclic conditioning with subsequent destructive testing. For a conservative estimate, a total of 20 loading and unloading cycles up to a 3% elongation and then a further 20 loading and unloading cycles up to 5% are performed. This is immediately followed by the quasi-static test until failure.

### 2.3. Sample Manufacturing

Two different manufacturing processes are examined: injection molded samples (hereinafter referred to as “injection molded”) and samples cut from an HDPE pipe manufactured using an extrusion process (hereinafter referred to as “pipe cut”). Pipe cut samples were provided by the manufacturer (“pipe cut manufacturer”) on the one hand and produced from offcuts (“pipe cut RWTH”) on the other. All injection molded samples were provided by the manufacturer.

All mechanical tests are carried out using uniaxial tensile tests on a dog bone specimen in accordance with ISO 527-2 [8].

### 2.4. Differential Scanning Calorimetry and Oxidation Induction

DSC is an analytical measurement method used to investigate the thermal properties of materials, such as phase transitions [43]. It is usually carried out in an inert gas atmosphere to prevent oxidative decomposition reactions in polymers. However, if the oxidation stability of a polymer or the quantity (quality) of the added stabilizers is to be determined, the oxidation induction time (OIT) can be used, which is also determined using DSC measurements. In this process, heating is first carried out in the nitrogen flow. When the specified temperature is reached, the atmosphere is replaced by the oxygen atmosphere at the same flow rate. The test specimen is then held at a constant temperature until the oxidation reaction is indicated by an exothermic deviation of the DSC heat flow curve.

It is known from the literature from measurements of the oxidation induction temperature that the start of the oxidation of HDPE can be above 250 °C [44]. However, temperatures from 200 °C are also found for polyethylene itself. In this article, 200 °C was therefore chosen as the test temperature in order to achieve the best possible resolution of the various OITs. Tests were performed with a *DSC 214 Polyma* by NETZSCH-Gerätebau GmbH, Germany.

## 3. Results

### 3.1. Mechanical Behavior and Surface Conditions

**Figure 9:**
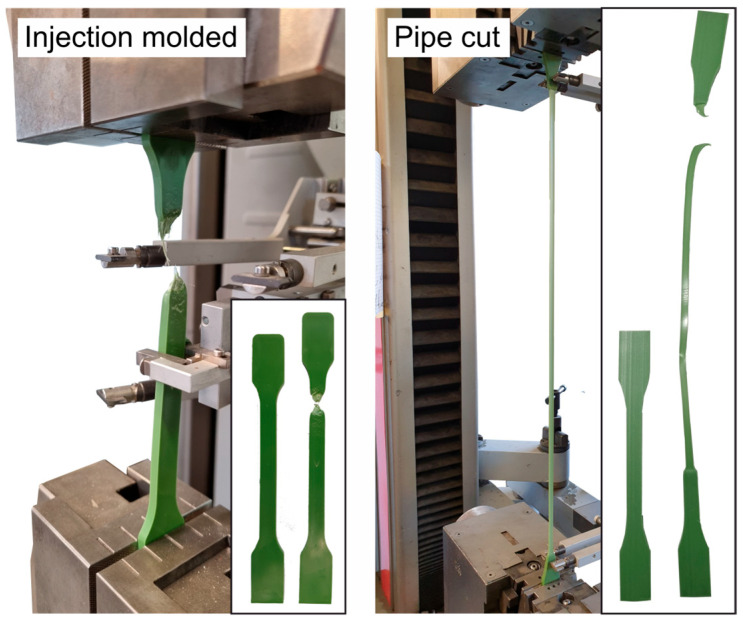
Comparison of the deformation and failure behavior of injection molded and extruded specimens.

The comparison of the mechanical values shown in Table 3 clearly shows the influence of sample production on the mechanical behavior. Compared to extruded samples, injection molded samples have virtually no yield potential. After reaching the maximum stress, failure occurs relatively quickly. The sample tapers considerably before a crack forms, which then leads to cross-sectional failure. In the case of extruded samples cut from the HDPE pipe, on the other hand, a pronounced yielding of the material can be seen, which is accompanied by a cross-sectional tapering that occurs over the entire gauge length (Figure 9).

**Figure 10:**
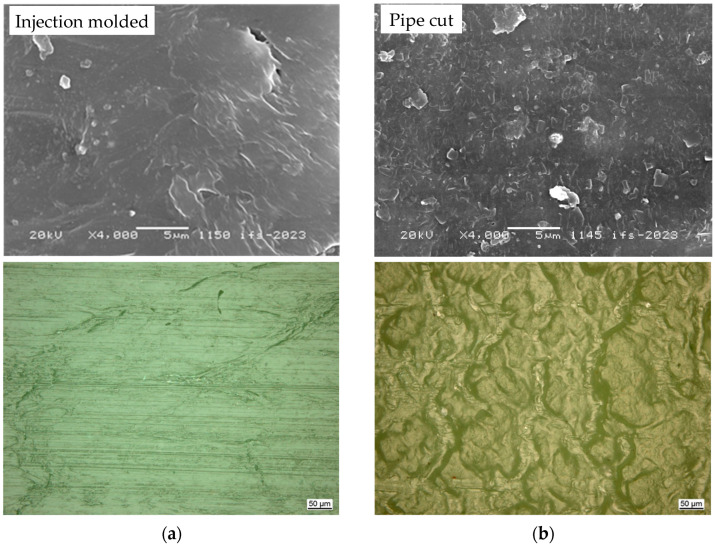
Comparison of SEM (top) and reflected light microscope images (bottom) of (**a**) injection molded and (**b**) extruded samples.

The influence of the manufacturing process with different cooling processes and the differences resulting from the formation of different superstructures can also be clearly seen in images taken using a scanning electron microscope (SEM) and reflected light microscope, Figure 10. Compared to the extruded samples, the surface texture of the injection molded samples is much smoother. The extruded samples have a granular-structured surface, which indicates a bigger spherulite formation.

### 3.2. Sanding Method

**Figure 11:**
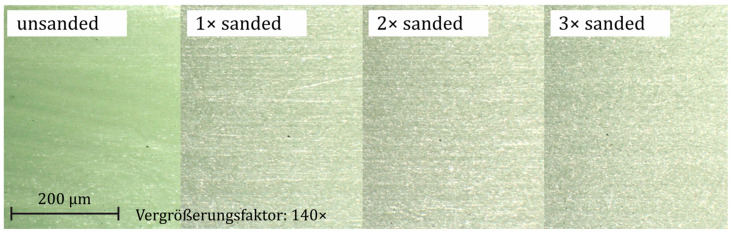
Microscope images of differently sanded samples (injection molding).

Irradiating the samples with quartz sand damages the surface of the material. The microscope images shown in Figure 11 reveal a clear difference between the unsanded and sanded sample surfaces. There are more light-colored, punctual defects and scratches, which can negatively influence the strength.

Additional sanding cycles lead to an optical homogenization of the surface. Therefore, the samples are only sanded once to avoid homogenization.

**Figure 12:**
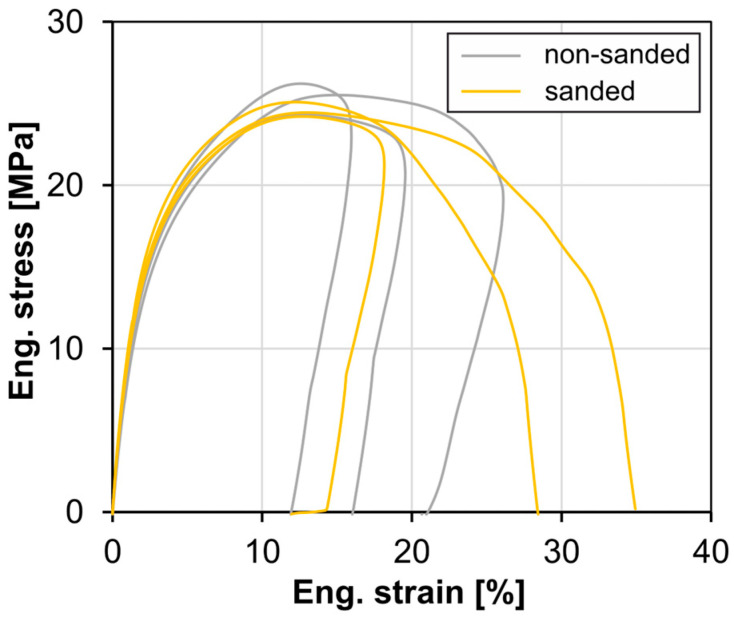
Tensile stress–strain curves of sanded and unsanded samples (injection molded).

A series of tests carried out on injection molded, sanded samples show no significant influence of sanding on the tensile strength, fracture strain or modulus of elasticity, Figure 12. However, since a damaged surface can influence degradation by increasing the area of attack during the accelerated aging process, sanding is carried out on a large number of samples.

### 3.3. Cyclical Conditioning

**Figure 13:**
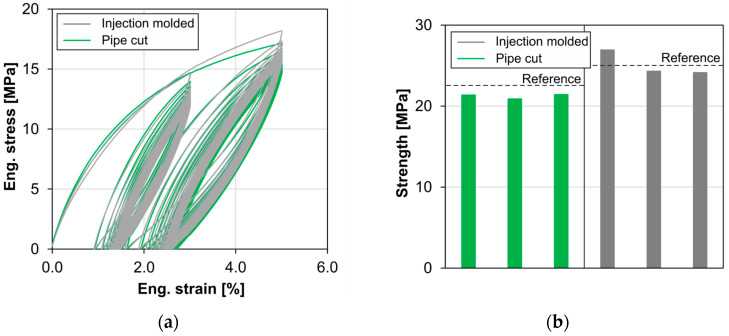
(**a**) Tensile stress–strain curves of cyclic conditioning (pipe cut and injection molded); (**b**) subsequently determined tensile strengths (pipe cut and injection molded, right).

The cyclic conditioning of the samples causes the formation of hysteresis curves (Figure 13). Due to the viscoelastic behavior of HDPE, a residual strain remains after returning to the force zero point, which increases degressively with increasing cycles. This behavior is evident for both strain levels investigated. There is no difference between injection molded and extruded samples. Overall, the behavior of both sample types in the linear-elastic range is almost identical, which also demonstrates the good agreement of the hysteresis curves.

After completion of the cycle runs at 3% and 5% elongations, the samples were visually inspected for localized defects and compared with control images of the unloaded condition. No defects or indications of damage were found.

The tensile strengths determined in subsequent quasi-static tests are reduced by approx. 1 MPa for the pipe cut samples compared to the non-conditioned reference samples (Figure 13b). The average strength of the injection molded samples, on the other hand, is at the level of the corresponding reference samples. The moduli of elasticity and the fracture strain show no irregularities in comparison to non-conditioned samples, considering the manufacturing process and the scattering.

### 3.4. Temperature Tests

**Figure 14:**
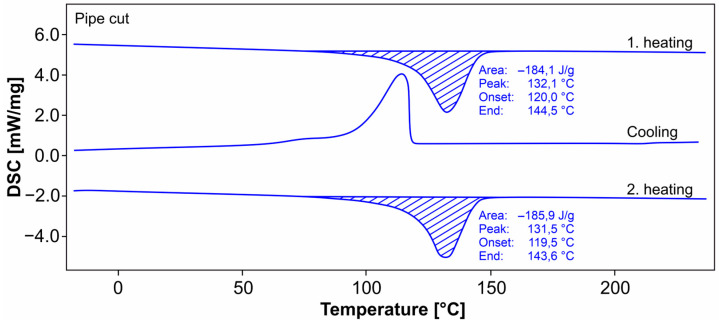
DSC analysis (pipe cut).

**Figure 15:**
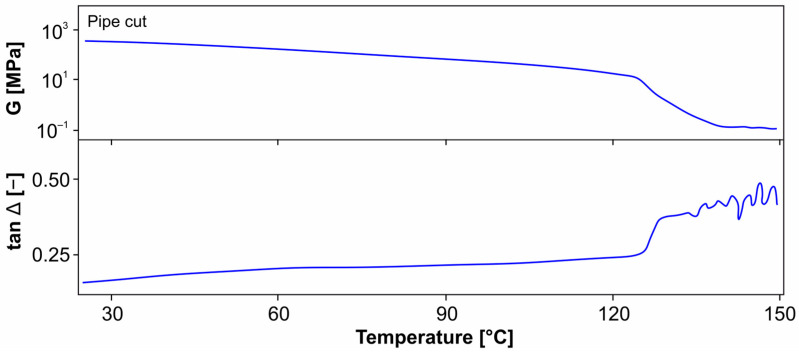
DMTA analysis (pipe cut).

The mechanical behavior of thermoplastics is strongly dependent on the temperature. In the differential thermal analysis (DSC) of the pipe cut material, Figure 14, there are still slight differences in the melting peak in the first heating due to the material history of the individual sample; in the second heating, a peak at 131 °C is obtained in both measurements. The comparative dynamic mechanical thermal analysis (DMTA), Figure 15, shows a melting point at 125 °C (evaluation of the tan Δ) with a constantly slightly decreasing shear modulus. The crystallization range ends in the DSC when cooling down to approx. 60 °C; the melting range of the first micro crystallites starts when heating up from 70 °C.

Using the average enthalpy of fusion of two samples with ΔH_m_ of 189.3 J/g and ΔH_m∞_ of 287.3 J/g at 100% crystallinity of PE results, according to Mirabella and Bafna [45], in a degree of crystallinity of 65.9% (and 65.3% using ΔHm_∞_ of 290 J/g like in [14]) for the pipe cut material used, with α = ΔH_m_/ΔH_m∞_ × 100%.

**Figure 16:**
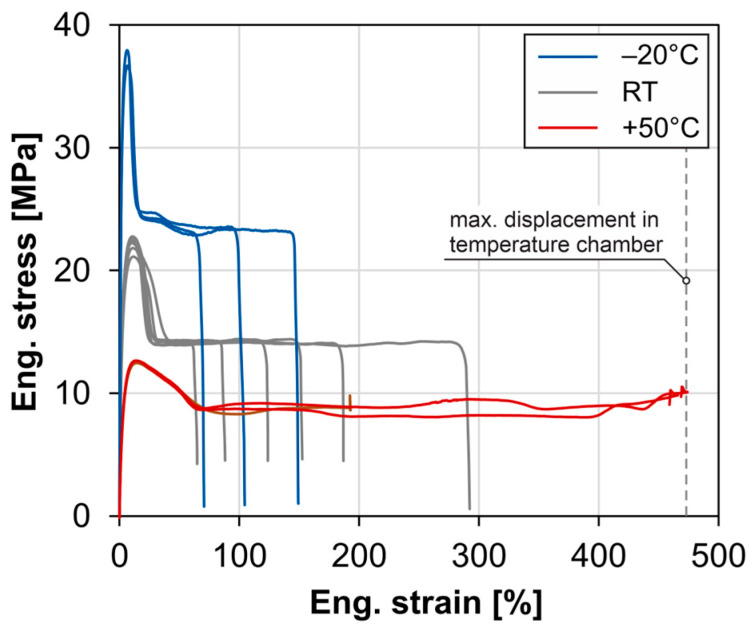
Stress–strain curves of the temperature tests (pipe cut).

**Figure 17:**
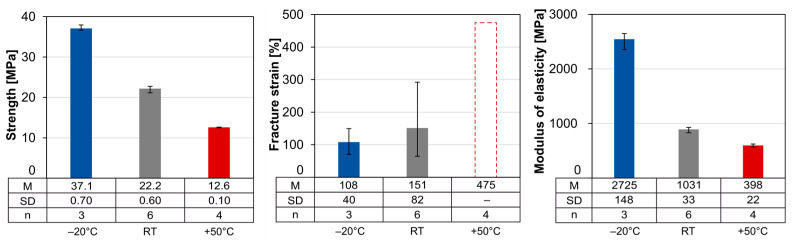
Average strength, fracture strain and modulus of elasticity of the temperature tests (pipe cut).

A comparison of the tensile stress–strain curves shown in Figure 16 illustrates the influence of test temperatures well below the melting range on the material. Figure 17 compares the material characteristics.

Poisson’s ratio is determined at room temperature and lies in a range from 0.40 to 0.45, depending on the evaluation method.

According to fib Bulletin [7], requirements for strength at RT and fracture strain at −20 °C and RT are specified for the pipe material. The requirements for strength and fracture strain at −20 °C are met, but the required minimum fracture strain is not met at RT.

### 3.5. Accelerated Aging

The results of the various accelerated aging methods are compared below. For reasons of clarity, the individual tensile stress–strain curves are not shown. Initially, only the results of the extruded pipe cut samples are shown.

**Figure 18:**
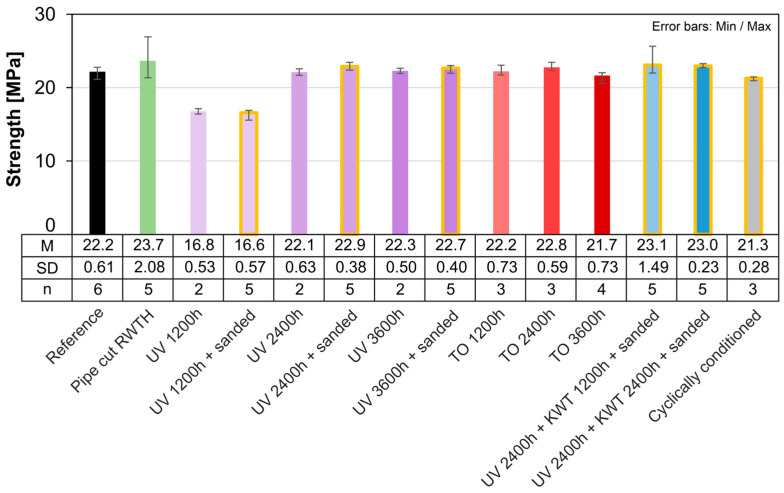
Comparison of the strength of artificially aged samples (pipe cut).

Considering the tensile strength first, it can be seen that the average strengths determined are between 21.3 MPa and 23.7 MPa (Figure 18). Only the samples from the 1200 h UV exposure are significantly below the other average strengths, with a strength of 16.8 MPa and 16.6 MPa, respectively. After a further 1200 h, the strength of the unaged samples can be observed again. An additional 1200 h then has no further influence on the strength. The experimental investigations were unable to provide reliable evidence to explain this phenomenon. However, various polymer–chemical effects are conceivable, which initially lead to debonding and subsequent re-polymerization. These include, for example, the destruction of chain bonds by UV radiation and the formation of free oxygen radicals (photodegradation). With increasing durations of UV irradiation, opposing effects initiated by the UV light, such as the formation of oxidative groups or the post-crosslinking of polymer chains, can lead to renewed polymerization. It is also conceivable that internal stresses are reduced during initial storage or that moisture is absorbed. Due to a lack of sample material, however, this could not be investigated further analytically. From the literature, this effect could not be observed in the pure temperature storage at 125 °C (i.e., without UV and without the influence of moisture) of thermoplastic blends made of LLDPE/HDPE. Here, there was only a reduction in tensile strength over a 210-day observation period [46]. In comparison, the long-term storage of HDPE geogrids at 25 °C, 45 °C and 75 °C in leachate for up to one year shows no changes in tensile strength [47]. However, this study shows an increase in fracture strain over time when stored in leachate at 75 °C.

In the present study, oven storage at +60 °C only had a marginal effect on the strength. Only after 3600 h is the average strength reduced by approx. 1 MPa and is therefore outside the scatter band of the pre-series (TO 2400 h). The effect of thermal oxidation is discussed in more detail in the context of differential scanning calorimetry (DSC) (see Section 3.9).

The aging test (KWT), on the other hand, does not lead to a reduction in strength. After cyclic conditioning, the strength is slightly reduced but is still within the scatter band of the reference samples. Sanded samples show a slightly increased strength compared to non-coated samples of the same series.

The evaluation of the fracture strain of extruded pipe cut samples shows an extreme volatility in the determined data. The lowest fracture strain is 55%, the highest value 700%. The enormous scatter that can be observed within series and across series makes it impossible to compare the various accelerated aging processes. As the geometric deviations between the samples are very small and the test and storage conditions within the series are identical, the cause of the high scatter can only be assumed to be in the material itself. For example, samples may have different local defects and degrees of crystallization and superstructures, which influence the yield capacity and thus also the fracture strain achieved. An indication of this would be the deviating melting behavior in the first heating run [44]. Since only a very limited number of samples were available, it was not possible to carry out comparative measurements on untested samples.

**Figure 19:**
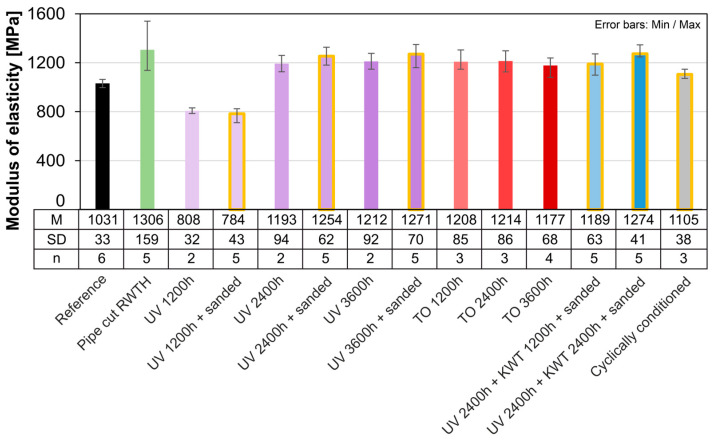
Comparison of the modulus of elasticity of artificially aged samples (pipe cut).

The similarity between the moduli of elasticity of different aging processes largely corresponds to the distribution of their strengths, but overall, all aging processes result in an increase in modulus compared to the reference (Figure 19). Nevertheless, the reduction in the average modulus after 1200 h of UV irradiation can also be observed here. After a further irradiation period of 1200 h, it increases again and is at the level of the other series. The cyclic conditioning results in a reduced modulus of elasticity compared to the other aging scenarios.

The strength requirements according to fib Bulletin [7] are met for all the accelerated aging processes investigated. The requirements for minimum fracture strain are only partially met due to the very high scatter.

**Figure 20:**
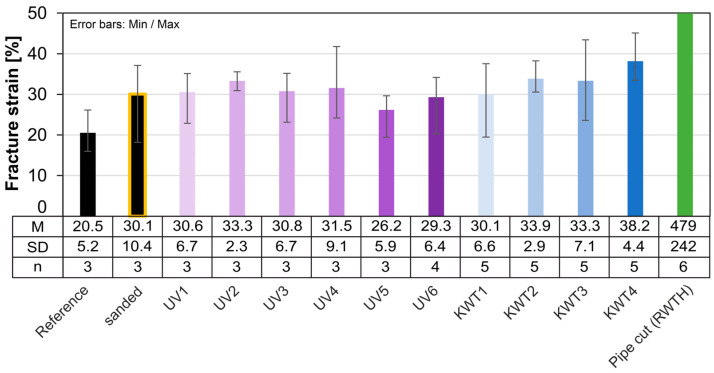
Comparison of the strength of artificially aged samples (injection molded).

Additional aging tests are carried out with further injection molded samples. For UV irradiation with condensation water phases, these are carried out over a period of six weeks. Three samples are taken and tested every week. The aim here is to achieve a more precise resolution of the initial aging. The additional aging tests have a total duration of 14.5 weeks. For comparison purposes, the respective results of the extruded pipe cut are also shown (Figure 20).

As with the extruded samples, accelerated aging has no effect on the strength of the injection molded samples. This is approx. 24 MPa for all series tested. The scatter is also extremely small and lower than for the extruded samples.

**Figure 21:**
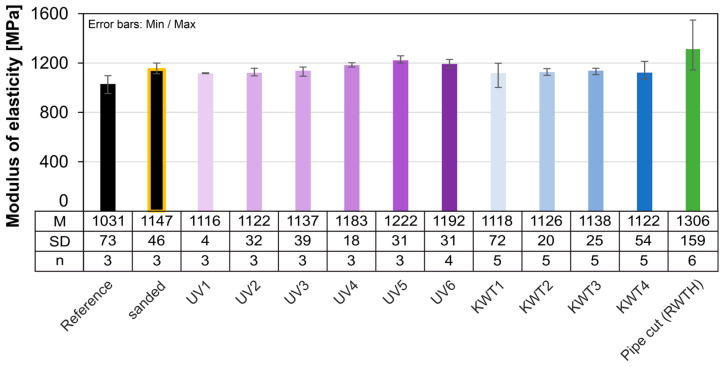
Comparison of the modulus of elasticity of artificially aged samples (injection molded).

As already discussed, the total fracture strain determined is much lower than for the extruded samples due to the lack of yield potential. However, the results here vary greatly, albeit less extremely than for the extruded samples. It is therefore not possible to make a statement regarding the effects of aging on the fracture strain. Only the aging test KWT shows a tendency towards an increase in the average fracture strain. The influence on the modulus of elasticity is also very low (Figure 21).

### 3.6. Compatibility

**Figure 22:**
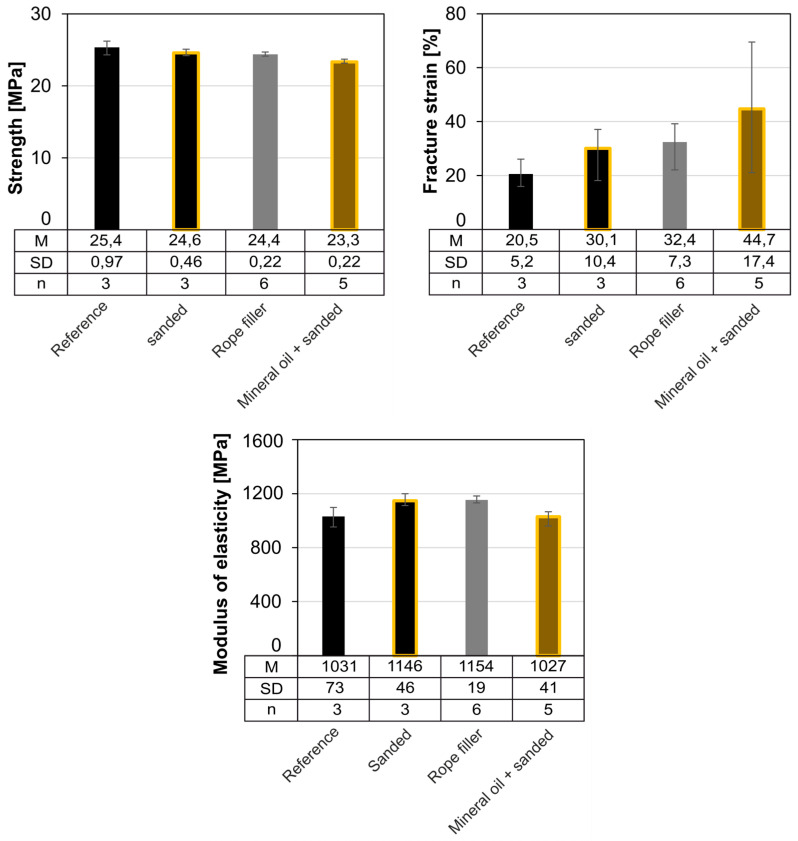
Average strength, fracture strain and modulus of elasticity of the compatibility tests (injection molded).

The results of the compatibility tests with rope filler and mineral oil (*TruLub A11*) are shown below (Figure 22). Storing samples in rope filler for fifty days reduces the average strength by 1 MPa. Storage in mineral oil also reduces the average strength by around 1 MPa compared to the sanded reference sample. At the same time, exposure to both substances cause an increase in fracture strain. However, the generally high scattering of this parameter should be noted here. Storage in the filling agent causes an increase of approx. 100 MPa, in mineral oil a reduction of approx. 100 MPa, of the modulus of elasticity. In principle, HDPE is resistant to mineral oils [48]. However, due to the immersion storage and a similar (non-)polarity of HDPE and mineral oil, it is conceivable that small quantities migrate into the HDPE and thus lead to the observed softening of the polymer.

No permanent discoloration occurred. No incompatibility with the cured WIRELOCK^®^ socketing compound was found.

### 3.7. Knife Cut and Groove

**Figure 23:**
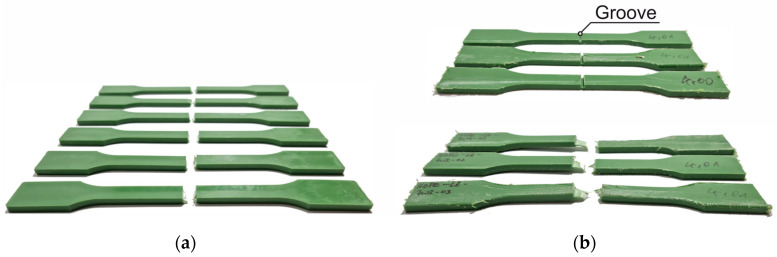
(**a**) Fracture patterns of the knife-edge samples; (**b**) sample and fracture patterns of the groove test.

During the intended service life and in particular during the installation of the bridge cables, defects in the HDPE sheathing can occur. In order to investigate the influence of notches and scratches on the load-bearing capacity, samples were provided with a measured cut orthogonal to the load direction. Three cutting depths of 0.5 mm, 1.0 mm and 1.5 mm were examined (Figure 23a). At this point, it should be noted that the thermoplastic properties of the polymer make it easy to repair surface defects.

In addition, samples were provided with a groove (width 2 mm, depth 2 mm) to simulate a larger cross-sectional recess (Figure 23b). Such a recess can be used, for example, for a force-fit connection of HDPE and potting compound within the rope sleeve.

**Figure 24:**
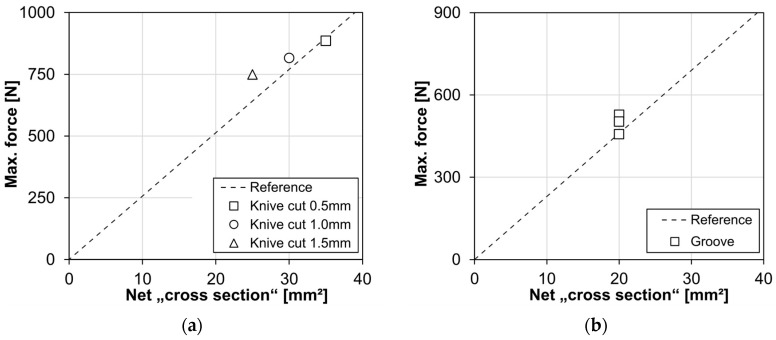
Influence of cross-section reductions on the load-bearing capacity: (**a**) knife cut (injection molded); (**b**) groove (pipe cut).

Both types of tests are intended to clarify whether cross-section defects lead to an excessive reduction in the load-bearing capacity of the HDPE compared to fully intact cross-sections. For this purpose, the maximum loads determined are plotted against the net cross-section (Figure 24). The reference curve shown is based on the reference samples without cross-sectional weakening. As all results are on or above the reference curve, it can be shown that the weakening of the cross-section does not lead to an excessive reduction in the load-bearing capacity or strength of the material.

### 3.8. Relaxation

The HDPE sheath has an extremely low stiffness compared to the bridge cables. As the HDPE is force-fitted to the outer Z-wire layer by extrusion, the deformation of the sheath corresponds to that of the rope. Therefore, creep tests were carried out to estimate the relaxation behavior of the polymer under imposed deformation. The two strain levels of 3% and 5%, which were already considered in the cyclic conditioning, were investigated. Injection molded samples were used. Since the selected strain levels are in the elastic range and this is virtually identical for injection molded and extruded samples, it can be assumed that the results are transferable to extruded HDPE.

**Figure 25:**
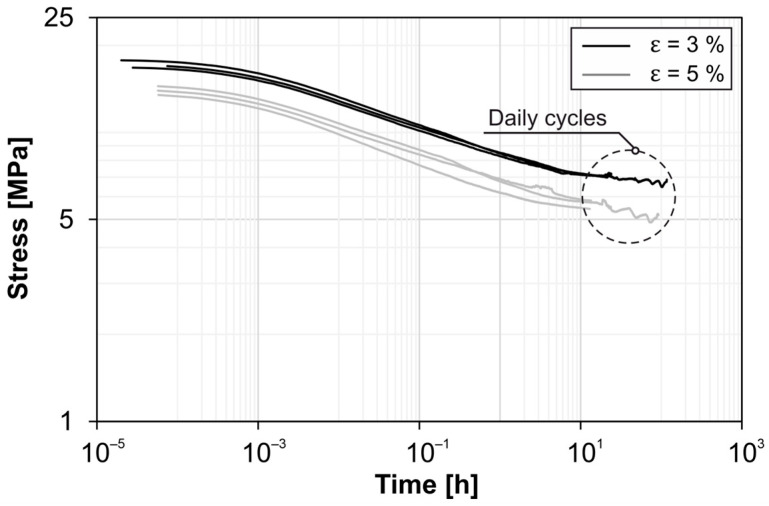
Relaxation tests with two different strain levels (injection molded).

The stress curves shown in Figure 25 demonstrate the viscoelastic character of the polymer. Under constant deflection, the stress decreases immediately. This is due to the movement and reorientation of the molecular chains [49] and a degressively decreasing process. A large part of the stress reduction takes place in the first few minutes after the deformation is imposed. After 12 h, the stress level for an applied strain of 5% has already fallen to 41.6% of the initial level; after 120 h, the material relaxes by only a further 0.3%. At this point, thermal effects already have a greater influence, as the results presented show. As the relaxation tests were not carried out at controlled temperatures, the jagged curves can be attributed to the different temperatures during the day and night. The reduction of the stresses and secant moduli can be seen in Table 4.

### 3.9. Differential Scanning Calorimetry and Oxidation Induction

Isothermal measurements at 200 °C were used to investigate the influence of oven storage and UV irradiation on the OIT of extrusion samples. The curves shown in the following figures represent the heat flow of the material over time. The OIT is reached when this changes significantly. A special tangent method is used to determine the point in time [50]. For a better overview, the curves, which actually overlap each other, are drawn apart.

**Figure 26:**
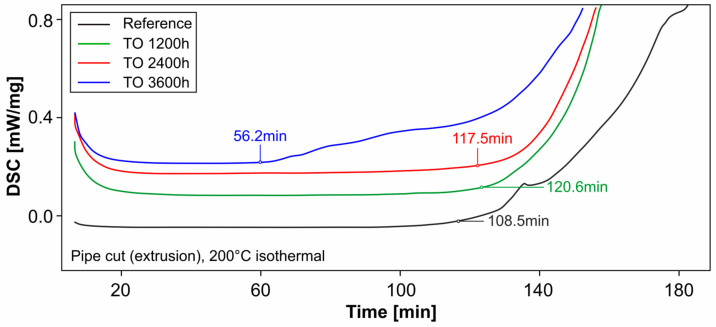
DSC: Influence of oven storage (pipe cut).

For oven storage at +60 °C, a significant reduction in OIT can only be seen for samples stored for 3600 h or 150 days (Figure 26). Compared to the reference samples, the duration decreases here by approx. 48%. The shorter storage times in the oven for 1200 or 2400 h show no influence here.

According to Arrhenius [51], chemical processes take place approximately twice to four times as fast when the reaction temperature is increased by +10 °C. If a reference temperature of 20 °C is selected and it is also taken into account that the test atmosphere of the DSC consists of 100% oxygen, this results in an oxidation stability of the HDPE pipes (reference, OIT = 108.5 min) in a normal atmosphere (21% oxygen) of approx. 258 years as a very rough estimation, without taking into account the effects of weathering, UV exposure and loss of stabilizers due to migration [52].

**Figure 27:**
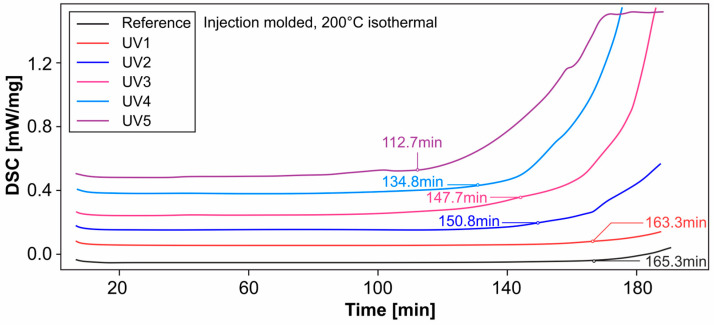
DSC: Influence of UV radiation (injection molded).

The results shown in Figure 27 show the influence of UV radiation in combination with moisture on the material. The test specimens are injection molded samples. The reference of the OIT corresponds to approx. one and a half times the reference of the OIT of the extrusion samples. The oxidation stability is continuously reduced by the artificial UV exposure and moisture. After five weeks, the effectiveness of the added antioxidants is still approx. 68% of the reference sample. However, it is still at a comparatively high level, higher than the reference samples of the pipe cut.

It should be mentioned at this point that reaching the OIT is not directly associated with a defect or loss of the mechanical, optical or chemical properties of the material. It merely indicates the point in time at which oxidation processes begin significantly. This point in time depends on the type and amount of stabilization of the material by the antioxidants. The influence on the mechanical properties cannot be derived directly from this.

## 4. Discussion

### 4.1. Manufacturing Process

The sample production was complex, as the HDPE coatings to be analyzed are extruded directly onto the ropes. The material tests had to be carried out before the ropes were manufactured, but industrial extrusion only takes place for the finished ropes with the corresponding color pigments and material thicknesses. For this reason, small batches of HDPE were injection molded, and a few samples were extruded in a small series prior to rope production, which then had to be compared with each other.

Our results show, in accordance with the literature [14,15], that the difference between the two manufacturing processes is particularly evident in the fracture strain achieved and in the yield behavior. This is most likely due to the cooling process of the molding compound during the respective manufacturing processes. In injection molding, the molecules have less time to orient and arrange themselves due to the rapid cooling within the surrounding mold, and the structures from crystallites and lamellae to spherulites are on nanoscale level. In contrast, the extrusion process allows for slower cooling, especially on the inner side of the material, and continuous movement of the polymer, which can lead to a different super structure due to bigger spherulites in microscale, as the SEM pictures show, and higher yield properties.

Due to the limited availability of samples cut from the pipe, not all aging processes can be investigated in the same way as with injection molded samples. However, as the strength and elongation at yield point of both sample types differs only slightly, it can be assumed that the influences on the material are comparable.

An important result with regard to the characterization of the material and the quality control of the product properties is the manufacturing process of the accompanying HDPE test specimens. Samples from injection molding did not exhibit any yield potential in the tests carried out here. Compared to pipe cut samples made from extruded material, the fracture strain is therefore many times lower. The average strength is at a similar level, but the modulus at a slightly reduced level compared to the extruded samples.

### 4.2. Accelerated Aging Tests

The influence of a variety of different accelerated aging processes on the material was also investigated. This proved to be very robust against all methods.

Parameters such as UV irradiation and temperature, water and salt had no significant influence on the strength and modulus of elasticity within a time frame of 3600 h for pipe cut samples. Compared to the results from the literature, a deterioration in the parameters would have been expected here. Even the influence of sand, which is blown onto the surface by wind and mechanically loads it, is not recognized in the mechanical characteristic values of our tests, even if the surface is clearly attacked. The reasons for this certainly lie in the experimental design. In many cases in the literature, especially in studies on the resistance of polymers, influencing variables are designed for the greatest possible damage. The influence of water/sea water in particular is often modeled using immersion, like in [22], which does not correspond to the application at hand here, as bridge cables always have the possibility of re-drying. There is also no permanent exposure to light (due to the night phases) like in [24], nor is the temperature permanently in high ranges like in [25]. The fact that a reduction in the characteristic values is visible in these literature contributions can be explained by the permanent exposure to the influencing factor. Due to the more realistic parameters we used, we do not come into a damage range. This emphasizes once again how important the selection of influences and the evaluation of results from accelerated aging tests are.

The service life should not be estimated at this point using the DSC-OIT. As also described in [26], misinterpretations can occur if a parameter (such as the oxygen partial pressure in the measuring system) does not correspond to the real influencing conditions when the test is carried out. However, the method is well suited to comparing a course, e.g., the need for stabilizers over time under different influences.

Compatibility with mineral oil and rope filler was demonstrated. When stored for 50 days in a mineral oil bath, the strength and modulus of elasticity are slightly reduced and fracture strain is slightly increased. This is most probably due to the migration of components of the oil into the HDPE. However, this is not considered to be critical, especially when considering the analysis in [39] in motor oil, because the sharp drop in yield strength of >30% in HDPE for water pipes after a storage period of 12 weeks or even much higher modulus losses in [15] for immersion in crude oil after one week exposure is not observed in our tests. No incompatibility with the WIRELOCK^®^ socketing compound was found either. We are unable to draw a comparison with results from the literature at this point, as this special filling material is not generally taken into account in compatibility studies in scientific articles.

Unfortunately, due to the limited material, we were not able to investigate the amount or migration of stabilizers and the separation of inner and outer layers (regarding, e.g., crystallinity, modulus) of the pipe sections in more detail. A detailed analysis of the oil absorption as in [15] in comparison to the structure and the mechanical parameters could have contributed further to our understanding and must be considered in further work. The same applies to the influence of moisture and UV exposure, as well as the determination of diffusion parameters. The low resolution of possible effects in our experiments, which are due to the inner and outer layer of the pipe sections and which would have to be separated individually, could be explained because in our case each sample represents the cross-section across the material, and differences can therefore be averaged out.

The mean fracture strain of our tests fluctuates very strongly in some cases. However, high scattering can be observed for all sample series including the reference series and regardless of the manufacturing process, so that the influence of accelerated aging is not a possible cause. As discussed in the section before, the causes for this are to be found in the material itself.

The reduction in strength and modulus of extruded samples after 1200 h of UV irradiation with condensation water phases is the only significant deviation from the other sample series. However, this effect could not be reproduced in a second study on injection molded samples. Due to the lack of sample material, no further analytical tests could be carried out.

In the opinion of the authors, other chemical environmental influences like solvents or cleaning agents are not decisive for the Fehmarnsund Bridge or comparable bridge structures.

Guo’s investigations have shown a Mullins effect due to shearing of the crystallites under cyclic loading [14]. In Figure 13, hysteresis behavior was observed under cyclic conditioning; this cannot be clearly attributed to the Mullins effect but can be explained by the viscoelastic material behavior. Further investigations would be necessary for this. However, here too there was no significant difference between pipe cut and injection molded samples in the linear-elastic range.

Overall, the results show the robust character and high weather resistance of the polymer material. The HDPE coating is a sensible alternative to the corrosion protection coatings previously used. As the HDPE is applied to the bridge cables at the factory, there is no need for a time-consuming on-site work step after the cables have been installed, which is a great advantage, especially in highly exposed locations. Local defects can be repaired with little effort due to the thermoplastic nature of the polymer. By adding appropriate colorants, the bridge cables can be manufactured in any color.

### 4.3. Influence of Climate Zones and Temperatures

The mechanical behavior of HDPE at different temperatures has been investigated, for example, in the literature sources [26,27,28,29] in Chapter 1.2.2, but mainly on HDPE pipes or geomembranes. The investigation of the HDPE sheathing of bridge cables has so far only been examined very sporadically and with a focus on specific influences [31,33,36,37]. In this article, we have attempted to take into account all the main influences that affect the sheathing of the cables in a bridge structure in order to draw a full picture. In the authors’ opinion, there is generally no reason why HDPE should not be used in different climatic zones; HDPE can even be used in very hot or very cold regions, taking into account the change in mechanical properties, see Section 3.4. The effectiveness of HDPE as corrosion protection is not affected by the temperatures.

The presented investigations were carried out during the planning of the cable replacement of the Fehmarnsund Bridge. The cable replacement is necessary to extend the service life of the bridge and to adapt the bridge to new conditions. Therefore, the selected aging regimes, temperatures and UV loads are adapted to the specific location. However, due to the exposed maritime location, these are very harsh corrosive conditions that do not apply to many bridges in Europe. Therefore, this is a component-related analysis, but the results can be transferred with caution to similar bridge structures. However, the level of UV exposure would have to be re-evaluated for other structures.

This point addresses the fundamental discussion of the transferability of accelerated aging tests in the construction industry.

### 4.4. Exceptional Effects

The behavior of the material was not investigated under extreme conditions such as an explosion or an impact/accident (increased temperatures + dynamic shock load), as this can be ruled out due to the roadway layout and bridge construction.

While the present investigations are tailored to the Fehmarnsund Bridge, the conceptual approach of the investigations can be transferred to other bridges in other climate zones at any time. It can be seen that the sample production has a strong influence on the results, and therefore the transferability to other production processes is limited. In addition, it is imperative that samples for reliable results are taken from original components.

## 5. Conclusions and Outlook

Full-locked coil ropes are provided with a multi-layered, epoxy resin and polyurethane-based corrosion protection coating in accordance with the state of the art according to ZTV-ING. This is applied after installation of the bridge cables and must be renewed at regular intervals. An alternative, innovative option is the use of an HDPE coating, which is extruded onto the rope during production. The material is also already being used for strand bundle ropes, where HDPE tubes are applied during assembly. HDPE-sheathed FLCRs have so far only been used on two bridges in Germany and require a certificate of applicability from the building authorities.

The mechanical behavior of the thermoplastic HDPE used for the cladding was comprehensively characterized on the basis of a large number of tests. Various accelerated aging procedures were derived based on the general conditions of the Fehmarnsund Bridge. The material proved to be robust against all the aging regimes carried out and showed no incompatibilities with substances with which it could come into contact during its service life. However, the method of sample production has a decisive influence on the yield behavior of the plastic.

Due to its resistance to climatic influences, the HDPE coating is a competitive alternative to conventional corrosion protection coatings for FLCRs.

## Figures and Tables

**Table 1:** Aging regime sequence from climate change test PV1200 (−20 °C/+50 °C) and salt spray test PV1210.

Test Cycles	Duration	Stages	Conditions
3 weeks modified aging test PV1200	1 h	Heating phase	+50 °C, 80% RH
	4 h	Cooling phase	+50 °C, 80% RH
	2 h	Cooling phase	−20 °C, from T < 0 °C non-regulated RH
	4 h	Holding phase	−20 °C, 80% non-regulated RH
	1 h	Heating phase	+23 °C, ab T = 0 °C r. L 30%
1 week salt spray test PV1210	4 h	Salt spray	
	4 h	Ambient climate	
	16 h	Condensing water climate, 40 °C	
3 weeks modified aging test PV1200	1 h	Heating phase	+50 °C, 80% RH
	4 h	Cooling phase	+50 °C, 80% RH
	2 h	Cooling phase	−20 °C, from T < 0 °C non-regulated RH
	4 h	Holding phase	−20 °C, 80% non-regulated RH
	1 h	Heating phase	+23 °C, ab T = 0 °C r. L 30%

**Table 2:** Overview of sample series and accelerated aging procedure.

Test Series			Accelerated Aging Procedures
	sanded	Non-sanded	
Manufacturing: Extrusion			
Reference	–	X	Non-aged reference samples
Pipe cut RWTH	–	X	Samples cut from HDPE pipe at the RWTH
UV 1200 h	X	X	UV irradiation + condensation, duration: 1200 h/50 d
UV 2400 h	X	X	UV irradiation + condensation, duration: 2400 h/100 d
UV 3600 h	X	X	UV irradiation + condensation, duration: 3600 h/150 d
TO 1200 h	–	X	Oven storage at +60 °C, duration: 1200 h/50 d
TO 2400 h	–	X	Oven storage at +60 °C, duration: 2400 h/100 d
TO 3600 h	–	X	Oven storage at +60 °C, duration: 3600 h/150 d
UV 2400 h + KWT 1200 h	X	–	UV irradiation + condensation, duration: 2400 h/100 d +
UV 2400 h + KWT 2400 h	X	–	Aging test PV1200 & PV1210, duration: 1200 h/50 d
Cyclically conditioned	X	-	UV irradiation + condensation, duration: 2400 h/100 d +
Manufacturing: Injection molding			
Reference	X	X	Non-aged reference samples
UV 1	–	X	UV irradiation + condensation, duration: 7 d
UV 2	–	X	UV irradiation + condensation, duration: 14 d
UV 3	–	X	UV irradiation + condensation, duration: 21 d
UV 4	–	X	UV irradiation + condensation, duration: 28 d
UV 5	–	X	UV irradiation + condensation, duration: 35 d
UV 6	–	X	UV irradiation + condensation, duration: 42 d
KWT 1	–	X	21 d KWT (−20/+50 °C 80% RH)
KWT 2	–	X	21 d KWT (−20/+50 °C 80% RH) + 2 d NK + 5 d PV1210 (salt spray test)
KWT 3	–	X	21 d KWT (−20/+50 °C 80% RH) + 2 d NK + 5 d PV1210 (salt spray test) + 2 d NK + 21 d KWT (−20/+50 °C 80% RH)
KWT 4	–	X	2 cycles: 21 d KWT (−20/+50 °C 80% RH) + 2 d NK + 5 d PV1210 (salt spray test) + 2 d NK + 21 d KWT (−20/+50 °C 80% RH)
Rope filler	–	–	Storage in rope filler, duration: 1200 h/50 d, RT
Mineral oil	X	–	Storage in mineral oil, duration: 1200 h/50 d, RT

KWT: aging test modified PV1200, NK: normal climate, RH: relative humidity.

**Table 3:** Comparison of the mechanical characteristic values between injection molding and pipe cut sample types.

		Tensile Strength [MPa]	Fracture Strain [%]	Modulus of Elasticity [MPa]
Injection molded	M	25.4	20.5	1031
	SD	0.97	5.2	73
Pipe cut manufacturer	M	22.2	151	1031
	SD	0.61	82	33
Pipe cut	M	23.7	479	1306
	SD	0.28	242	159

**Table 4:** Tensile stress and secant modulus as a function of the test time.

	t = 0 h		t = 12 h			t = 24 h			t = 120 h		
ε	σ_0_	E	σ	σ/σ_0_	E	σ	σ/σ_0_	E	σ	σ/σ_0_	E
[%]	[MPa]	[MPa]	[MPa]	[%]	[MPa]	[MPa]	[%]	[MPa]	[MPa]	[%]	[MPa]
3.0	13.97	466	5.68	40.6	189	5.41	37.4	180	5.19	35.9	173
5.0	17.17	343	7.14	41.6	143	7.10	41.4	142	7.05	41.1	141

## Data Availability

The original contributions presented in this study are included in the article. Further inquiries can be directed to the corresponding author(s).

## References

[1] Friedrich H., Hamme M., Kuhlmann U. (2021). Brückenseile. Stahlbau Kalender 2021.

[2] Bundesministerium für Digitales und Verkehr: ZTV-ING Teil 4: Stahlbau, Stahlverbundbau, Abschnitt 5: Korrosionsschutz von Brückenseilen. https://technical-regulation-information-system.ec.europa.eu/en/notification/26195/text/D/DE.

[3] Feyrer K., Wehking K.-H. (2017). FEYRER—Drahtseile, Bemessung, Betrieb, Sicherheit.

[4] Friedrich H. (2014). Bridge Ropes for Road Bridges—Comparison Between Fully Locked Coil Ropes and Bundles of Parallel Strands. Berichte der Bundesanstalt für Straßenwesen, Heft B 98 Brücken- und Ingenieurbau. http://bast.opus.hbz-nrw.de/volltexte/2014/767/pdf/B98b.pdf.

[5] (2010). Design of Steel Structures—Part 1-11: Design of Structures with Tension Components.

[6] Bundesministerium für Digitales und Verkehr: Technische Lieferbedingungen und Technische Prüfvorschriften für Ingenieurbauten. TL/TP-ING (2022) TL/TP-VVS, Part 4 Section 4 ‘Technical Delivery Conditions and Technical Test Specifications for Fully Locked Bridge Cables’. https://www.bast.de/DE/Publikationen/Regelwerke/Ingenieurbau/Baudurchfuehrung/TL_TP-Gesamt.pdf.

[7] (2005). fib Bulletin No. 30. Acceptance of Stay Cable Systems Using Prestressing Steals.

[8] (2012). Plastics—Determination of Tensile Properties—Part 2: Test Conditions for Moulding and Extrusion Plastics.

[9] Millfield Enterprises (Manufacturing) Limited (2023) Wirelock—Technical Data Manual; V10-1123. https://www.wirelock.com/.

[10] (2022). Terminations for Steel Wire Ropes—Safety—Part 4: Metal and Resin Socketing.

[11] Ehrenstein G. (2011). Polymer Werkstoffe, Struktur—Eigenschaften—Anwendungen, 3. Auflage.

[12] Ehrenstein G., Pongratz S. (2007). Beständigkeit von Kunststoffen. Band 1.

[13] Elleuch R., Taktak W. (2006). Visocelastic behavior of HDPE Polymer using Tensile and Compressive Loading. J. Mater. Eng. Perform..

[14] Guo H., Rinaldi R.G., Tayakout S., Broudin M., Lame O. (2021). Characterization of the spherulitic deformation in equatorial region and cavitation in HDPE materials submitted to mixed-mode oligo-cyclic tensile loading. Polym. Test..

[15] Chaoui K., Ghabeche W., Azzouz S. Study of Crude Oil Interaction with High Density Polyethylene Pipe Surface Material. 24ème Congrès Français de Mécanique. https://www.researchgate.net/publication/335994735_STUDY_OF_CRUDE_OIL_INTERACTION_WITH_HIGH_DENSITY_POLYETHYLENE_PIPE_SURFACE_MATERIAL.

[16] (2023). Plastics—Determination of Charpy Impact Properties—Part 1: Non-Instrumented Impact Test.

[17] (2015). Thermoplastics Pipes—Determination of Tensile Properties—Part 1: General Test Method.

[18] (2015). Thermoplastics Pipes—Determination of Tensile Properties—Part 3: Polyolefin Pipes.

[19] (2018). Standard Test Method for Density of Plastics by the Density-Gradient Technique.

[20] (2013). Standard Test Method for Melt Flow Rates of Thermoplastics by Extrusion Plastometer.

[21] Albozahid M.A., Diwan A.A., Diwan M.A., Alansari L.S. (2021). Effect of Weathering on Tensile Properties of Low and High Density Polyethylene. IOP Conf. Ser. Mater. Sci. Eng..

[22] Elkori R., Lamarti A., Salmi H., El Had K., Hachim A., Yamari I. Stady of the Effekt of Accelerated Aging by Sea Water on the Mechanical and Chemical Properties of High Density Polyethylene Bottles. https://www.researchsquare.com/article/rs-1893605/v1.

[23] Hsueh H.-C., Kim J.H., Orski S., Fairbrother A., Jacobs D., Perry L., Hunston D., White C., Sung L. (2020). Micro and macroscopic mechanical behaviors of high-density polyethylene under UV irradiation and temperature. Polym. Degrad. Stab..

[24] Carrasco F., Pages P., Pascual S., Colom X. (2001). Artificial aging of high-density polyethylene by ultraviolet irradiation. Eur. Polym. J..

[25] Zhao B., Zhang S., Sun C., Guo J., Yu Y.X., Xu T. (2018). Aging behavior and properties evaluation of high-density polyethylene (HDPE) in heating-oxygen environment. IOP Conf. Ser. Mater. Sci. Eng..

[26] Mueller W., Jakob I. (2003). Oxidative resistance of high-density polyethylene geomembranes. Polym. Degrad. Stab..

[27] Ewais A., Rowe R.K. (2014). Effect of aging on the stress crack resistance of an HDPE geomembrane. Polym. Degrad. Stab..

[28] Rowe R.K., Ewais A. (2015). Ageing of exposed geomembranes at locations with different climatological conditions. Can. Geotech. J..

[29] Guermazi N., Elleuch K., Ayedi H.F. (2009). The effect of time and aging temperature on structural and mechanical properties of pipeline coating. Mater. Des..

[30] Saul R., Nützel O. (2014). Neuartige Sanierung der Tragkabel einer Hängebrücke in Norwegen. Stahlbau.

[31] Yu Y., Man M., Zhao F., Lin S., Guo F. (2020). Corrosive degradation evaluation of semi-parallel wire cables with high-density polyethylene sheath breaks. Eng. Fail. Anal..

[32] Huang R., Yu J., Yang Q., Chen J., Song Q. (2009). Discussion on the Wear Durability of HDPE Outer Sheath for Bridge Cable. Prestress. Technol..

[33] Liu S., Su H., Xu J., Zhou Z., Hao H., Wei L., Du J. (2024). Experimental study on tensile properties of HDPE sheath for parallel wire suspender after photo-oxidative aging. Constr. Build. Mater..

[34] Mkacher I., Brument Y., Murin V., Sellier I., Colin X. Methodology for evaluating the durability of HDPE outer sheaths of underground electric cables. Proceedings of the 21st International Conference on Electricity Distribution (CIRED).

[35] Lin B., Zhang C. (2022). Photo-aging performance of high-density polyethylene sheath in dry environment. Advances in Civil Engineering: Structural Seismic Resistance, Monitoring and Detection.

[36] Dan D., Cheng W., Sun L., Guo Y. (2016). Fatigue durability study of high density polyethylene stay cable sheathing. Constr. Build. Mater..

[37] Dan D., Sun L., Guo Y., Cheng W. (2015). Study on the Mechanical Properties of Stay Cable HDPE Sheathing Fatigue in Dynamic Bridge Environments. Polymers.

[38] Lai J., Bakker A. (1994). Analysis of the non-linear creep of high-density polyethylen. Polymer.

[39] Rezakalla A.I., Gennadyevech D.A. (2022). Effect of Certain Hydrocarbon Compounds on High-density Polyethylene Water Pipes. Mater. Plast..

[40] (1985). Testing of Glass and Plastics—Abrasion Test—Sand Trickling Method.

[41] (2016). Plastics—Methods of Exposure to Laboratory Light Sources—Part 1: General Guidance.

[42] (2016). Plastics—Methods of Exposure to Laboratory Light Sources—Part 3: Fluorescent UV Lamps.

[43] Grellmann W., Seidler S. (2015). Kunststoffprüfung.

[44] Qiao Z., Wu W., Wang Z., Zhang L., Zhou Y. (2022). Space Charge Behavior of Thermally Aged Polyethylene Insulation of Track Cables. Polymers.

[45] Mirabella F.M., Bafna A. (2002). Determination of the crystallinity of polyethylene/α-olefin copolymers by thermal analysis: Relationship of the heat of fusion of 100% polyethylene crystal and the density. J. Polym. Sci. Part B Polym. Phys..

[46] Lunzhi L., Jinghui G., Lisheng Z., Kai Z., Xiaohan Z. (2022). Aging phenomena in non-crosslinked polyolefin blend cable insulation material: In: Electrical treeing and thermal aging. Front. Chem..

[47] Kiersnowska A., Fabianowski W., Koda E. (2020). The Influence of the Accelerated Aging Conditions on the Properties of Polyolefin Geogrids Used for Landfill Slope Reinforcement. Polymers.

[48] Professional Plastics (2024) HDPE and LDPE Resistance Chart by Chemical. https://www.professionalplastics.com/professionalplastics/HDPE-LDPEChemicalResistanceChart.pdf.

[49] Rösler J., Harders H., Bäker M. (2019). Mechanisches Verhalten der Werkstoffe.

[50] (2023). Kunststoffe—Dynamische Differenzkalorimetrie (DSC)–Teil1: Allgemeine Grundlagen (ISO11357-1:2023); Deutsche Fassung EN ISO11357-1:2023.

[51] Binnewies M., Finze M., Jäckel M., Schmidt P., Willner H., Rayner-Canham G. (2016). Allgemeine und Anorganische Chemie.

[52] Troughton (2008). Handbook of Plastics Joining: A Practical Guide.

